# Risk factors for developing liver cancer in people with and without liver disease

**DOI:** 10.1371/journal.pone.0206374

**Published:** 2018-10-29

**Authors:** Jae Kyung Suh, Jayoun Lee, Jeong-Hoon Lee, Sangjin Shin, Ha jin Tchoe, Jin-Won Kwon

**Affiliations:** 1 Division of Healthcare Technology Assessment Research, National Evidence-based Healthcare Collaborating Agency (NECA), Seoul, Korea; 2 Department of Internal Medicine, Seoul National University Hospital, Seoul, Korea; 3 College of Pharmacy and Research Institute of Pharmaceutical Sciences, Kyungpook National University, Dae-gu, Korea; University of Cincinnati College of Medicine, UNITED STATES

## Abstract

**Background:**

The National Liver Cancer Surveillance Program (NLCSP) targets patients with liver diseases that lead to liver cancer in South Korea. This study aimed to investigate the risk of liver disease leading to liver cancer using nationally representative data to establish an efficient NLCSP.

**Methods:**

This study used data from the National Health Insurance Service National Sample Cohort (NHIS-NSC) from 2002 to 2013. A retrospective matched cohort design was applied to compare the development of liver cancer in patients with and without liver disease. Cox- proportional hazard regression for liver cancer with competing risk of death was performed for all subjects or each group stratified according to age or income level.

**Results:**

A total of 66,192 patients with liver disease and matched subjects without liver disease were included in the study. The incidences of liver cancer among patients with and without liver disease within a median 8-year follow-up period were 2.68% (n = 1,772) and 0.34% (n = 210), respectively. Cox- regression analysis for liver cancer incidence indicated that cirrhosis had the highest risk (hazard ratio [HR]: 18.13, 95% confidence interval [CI]: 15.24–21.58), followed by hepatitis B (HR: 9.32, 95% CI: 8.00–10.85). Subgroup analysis showed that the presence of liver disease was an important risk factor in younger as well as elderly people, and a higher risk of liver disease was also observed in the patients with Medicaid.

**Conclusions:**

Attention should be paid to the development of liver cancer in young people under 50 years old and preventive efforts to decrease the incidence of liver cancer among Medicaid recipients is needed.

## Introduction

Liver cancer is the sixth most common cancer worldwide, with approximately 780,000 new cases in 2012. Most of them, about 83%, occurred in less developed countries, and the highest incidence was observed in Asia and Africa [1]. The incidence trends of liver cancer differed worldwide. In the case of Asian and Pacific Islanders, the liver cancer incidence showed a steady or decreasing pattern recently [2]. In Korea, the overall liver cancer incidence increased from 1999 to 2014, but the total age-standardized incidence decreased steadily from 31.9 per 100,000 in 1999 to 19.9 per 100,000 in 2014. However, liver cancer is still the second most common cause of cancer-related death and the prognosis of hepatocellular carcinoma (HCC) worsens with the progression of cancer [3].

The risk factors for HCC accounting for 80% of liver cancers are well known, which include chronic hepatitis B virus (HBV) or hepatitis C virus (HCV) infection, certain metabolic liver diseases, and cirrhosis [4]. Perz et al. (2006) reported that HBV and HCV were the main causes of HCC. And more than 50% and 25% of HCC patients had HBV and HCV, respectively [5]. Although some patients with HBV infection progress to HCC without experiencing fibrosis or cirrhosis, approximately 85% of patients develop HCC through cirrhosis [6]. It is well known that HCC surveillance could improve the early detection and survival of HCC patients with HBV and HCV infection [7–9]. Asian countries such as Japan [10] and Korea [11] where the prevalences of HBV and HCV are relatively high have national surveillance programs. In South Korea, the National Liver Cancer Surveillance Program (NLCSP) provides screening tests including liver ultrasound at 6-month intervals for patients at high risk for developing HCC, such as men and women older than 40 years of age who have positive for hepatitis B surface antigen or anti-HCV or who have underlying liver cirrhosis. Individuals with income above the 50th percentile of National Health Insurance (NHI) beneficiaries are subsidized with 90% of the HCC surveillance cost whereas others (those with income level under the 50th percentile of NHI and Medicaid recipients) have no copayment [12]. In addition to the surveillance for high-risk adults, there is also a program for perinatal transmission prevention of hepatitis B. By implementing the program, perinatal infection has decreased in incidence, but it is still the major cause of hepatitis, which could progress to liver cancer.

The purpose of this study was to investigate the risk of liver disease progressing to liver cancer as well as clinical and socioeconomic risk factors for liver cancer using nationally representative data. The results of this study would support countries conducting or considering implementing an NLCSP in establishing an effective surveillance system.

## Methods

### Data source

This study was conducted using data from the National Health Insurance Service National Sample Cohort (NHIS-NSC), from 2002 to 2013 [13]. Raw data were available to the researchers upon reasonable academic request and with the permission of the Korean NHIS Institutional Data Access (http://nhiss.nhis.or.kr). The NHIS-NSC is a nationally representative sample obtained using systematic stratified random sampling based on the original claims database for the purpose of universal national insurance coverage. A total of 1,476 strata were derived based on age (n = 18), sex (n = 2), and income level based on type of insurance (n = 41). In 2002, 1,025,340 participants, comprising 2.2% of the total eligible Korean population, were selected and followed until 2013 unless a participant was disqualified due to death or emigration. The cohort contains four databases with the following information: insurance eligibility including information on socioeconomic variables; medical treatments with information from electronic medical treatment bills, details of disease and prescriptions; type and location of medical care institution and the number of beds; and general health examination results of the nationwide health examinations conducted by the NHIS from 2002 to 2013. The details of the cohort profile have been published [13] and has been used for academic research and decision making by policymakers to improve the health of the Korean population.

### Study population and design

This study had a retrospective matched cohort design to compare the development of liver cancer in patients with and without liver disease. Patients with liver disease were defined as those who had chronic hepatitis (International Classification of Diseases, Tenth Revision [ICD-10] code B18), HBV (ICD-10 code B18.0, B18.1), HCV (ICD-10 code: B18.2), cirrhosis (ICD-10 codes K70.2, K70.3, K74.x, and K71.7), or other liver diseases including alcoholic liver disease (ICD-10 codes K70, K70.1, K70.9, K76.1, K76.5, K76.6, and Z22.5) from 2003 to 2009. The cirrhosis group included all patients who were diagnosed with cirrhosis from any cause. Patients without liver disease were defined as those who were not diagnosed with any of the liver disease codes during the same period. The date of the first liver disease diagnosis was defined as the index date, and at least 1 year of history before the index date was used to identify newly diagnosed liver diseases. Patients who had a history of liver disease before the index date were excluded. We also excluded patients diagnosed with HCC within 1 year from the index date and patients who died before the index date. Each patient with liver disease was matched 1:1 with a corresponding patient without liver disease with respect to sex, age, and level of income.

### Main outcome measures

The primary outcome for this study was the incidence of liver. In addition, non-liver cancer-related mortality was investigated using competing risks in the statistical analysis. Each patient was followed from the index date to the date of the first diagnosis of liver cancer, death, or the end of the study period (December 31, 2013). As a proxy of active liver cancer, patients with liver cancer were defined as patients hospitalized with a diagnosis of malignant neoplasm of the liver and intrahepatic bile ducts (ICD-10 code C22). We identified non-liver cancer-related mortality as the death of a patient who had not been diagnosed with liver cancer during the study period.

### Covariates

We adjusted for comorbidities to control the confounding factors. Comorbidity was defined as a disease other than liver disease, or AIDS/HIV among the diseases included in the Charlson Comorbidity Index: myocardial infarction, congestive heart failure, peripheral vascular disease, cerebrovascular disease, dementia, chronic pulmonary disease, connective tissue disease-rheumatic disease, peptic ulcer disease, diabetes without complications, diabetes with complications, paraplegia and hemiplegia, renal disease, cancer, and metastatic carcinoma.

### Statistical analysis

Baseline comorbidities of the two groups, patients with liver disease and matched patients without liver disease, were compared based on descriptive statistics. Survival analyses in terms of time to develop liver cancer or death were conducted using the Kaplan-Meier method. We stratified the sample population by age group and income level based on type of insurance to evaluate the strength of the association of those risk factors with liver cancer in each subgroup. Cox proportional hazard regression for liver cancer development was performed for the entire study population and for each stratified group considering death (i.e., non-liver cancer-related mortality) as a competing risk. A competing risk is an event that can preclude the event of interest or affect the chance of the event occurring. Univariate and multivariate Cox regression analyses were used to assess the associations of liver cancer with factors of interest. To investigate the trends of liver cancer incidence and general mortality not related to liver cancer by age and income level in patients with and without liver disease, we conducted subgroup analysis of stratified age and income groups. The analyses were performed using SAS software version 9.2 (SAS Institute Inc., Cary, NC). This study was approved by the Institutional Review Board of National Evidence-based Healthcare Collaborating Agency (NECA IRB 16–003).

## Results

### Patient characteristics

**Fig 1:**
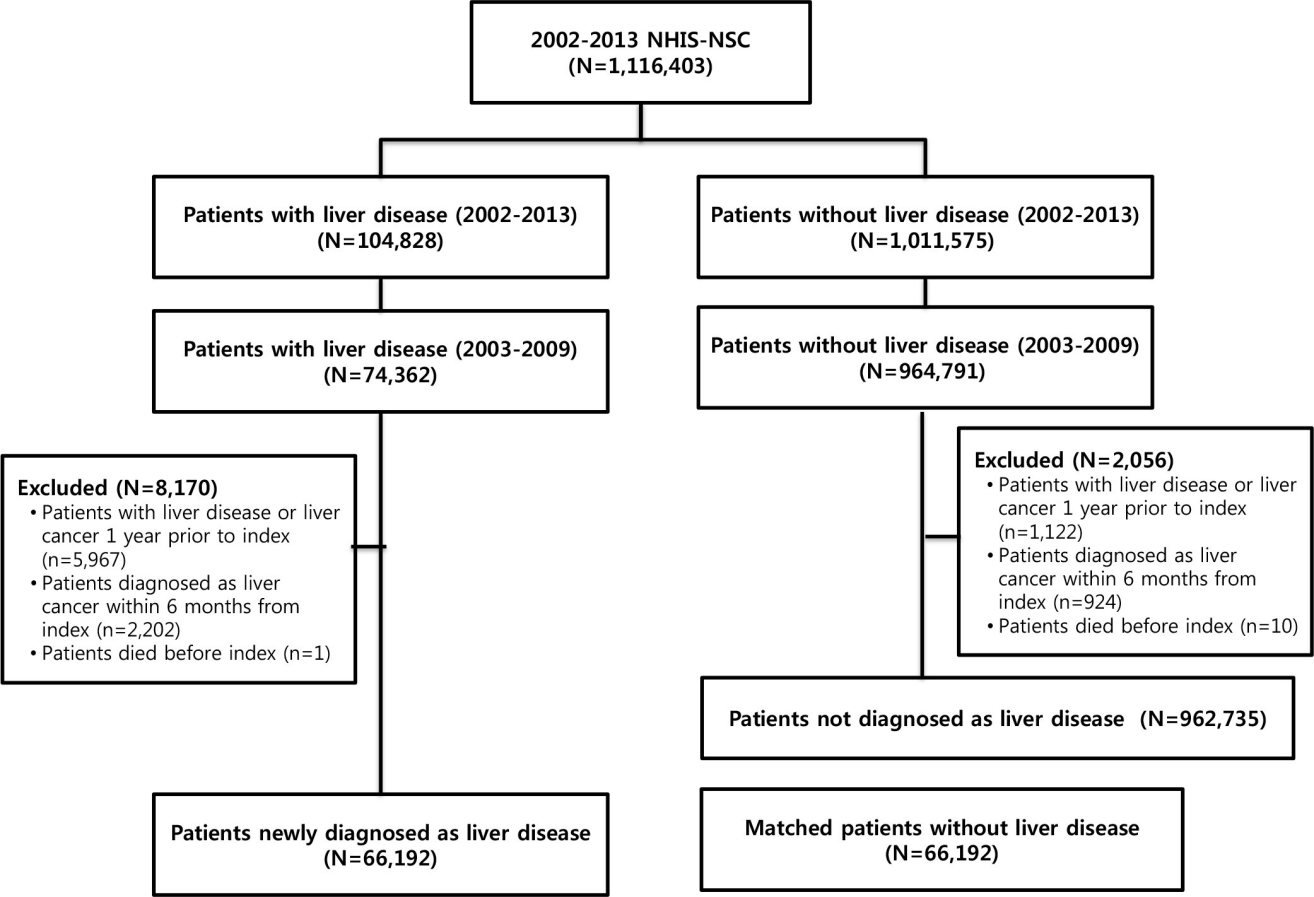
Patient flow chart representing the selection procedure based on the National Health Insurance Service National Sample Cohort (NHIS-NSC) dataset.

Among 1,116,403 subjects from the NHIS-NSC between 2002 and 2013, 66,192 patients with liver disease were included in the study and matched subjects without liver disease were identified (Fig 1). The demographic characteristics of the study population are presented in Table 1. Subjects were more likely to be men (63.54%), and more than 60% of the patients were 40 years of age or older (66.05%). Regarding the insurance type, the proportions of NHI district subscribers, NHI employee subscribers, and Medicaid recipients were 43.24%, 55.33%, and 1.44%, respectively. The most common comorbidity was peptic ulcer disease (21.90%), followed by diabetes (11.04%) in the exposure group. Similarly, peptic ulcer disease (9.25%) and diabetes (3.41%) were common in the non-exposure group, but their proportions were lower than in the exposure group (Table 1).

**Table 1:** Demographics of the study population.

		Patients with liver disease		Patients without liver disease	
		N = 66,192		N = 66,192	
		n	(%)	n	(%)
**Sex**	Men	42,061	(63.54)	42,061	(63.54)
	Women	24,131	(36.46)	24,131	(36.46)
**Age, years**	0–19	2,922	(4.41)	2,922	(4.41)
	20–29	7,141	(10.79)	7,141	(10.79)
	30–39	12,406	(18.74)	12,406	(18.74)
	40–49	16,647	(25.15)	16,647	(25.15)
	50–59	13,165	(19.89)	13,165	(19.89)
	60–69	8,993	(13.59)	8,993	(13.59)
	**≥**70	4,918	(7.43)	4,918	(7.43)
**Level of income based on type of insurance**	NHI district subscriber	28,620	(43.24)	28,620	(43.24)
	<50%	10,782	(16.29)	10,782	(16.29)
	**≥**50%	17,838	(26.95)	17,838	(26.95)
	NHI employee subscriber	36,621	(55.33)	36,621	(55.33)
	<50%	14,412	(21.77)	14,412	(21.77)
	**≥**50%	22,209	(33.55)	22,209	(33.55)
	Medicaid	951	(1.44)	951	(1.44)
**CCI**	Peptic ulcer disease	14,499	(21.90)	6,125	(9.25)
	Cancer	4,925	(7.44)	2,554	(3.86)
	Diabetes without complications	4,541	(6.86)	1,253	(1.89)
	Diabetes with complications	2,764	(4.18)	1,001	(1.51)
	Cerebrovascular disease	2,461	(3.72)	1,204	(1.82)
	Connective tissue disease-rheumatic disease	1,697	(2.56)	884	(1.34)
	Peripheral vascular disease	1,685	(2.55)	392	(0.59)
	Congestive heart failure	1,450	(2.19)	637	(0.96)
	Moderate or severe liver disease	561	(0.85)	25	(0.04)
	Renal disease	502	(0.76)	160	(0.24)
	Myocardial infarction	456	(0.69)	214	(0.32)
	Metastatic carcinoma	385	(0.58)	169	(0.26)
	Dementia	313	(0.47)	126	(0.19)
	Paraplegia and hemiplegia	202	(0.31)	123	(0.19)
	Chronic pulmonary disease	44	(0.07)	18	(0.03)
	AIDS/HIV	24	(0.04)	4	(0.01)

NHI: National health insurance; CCI: Charlson Comorbidity Index.

### Liver cancer incidence and non-liver cancer-related mortality

**Fig 2:**
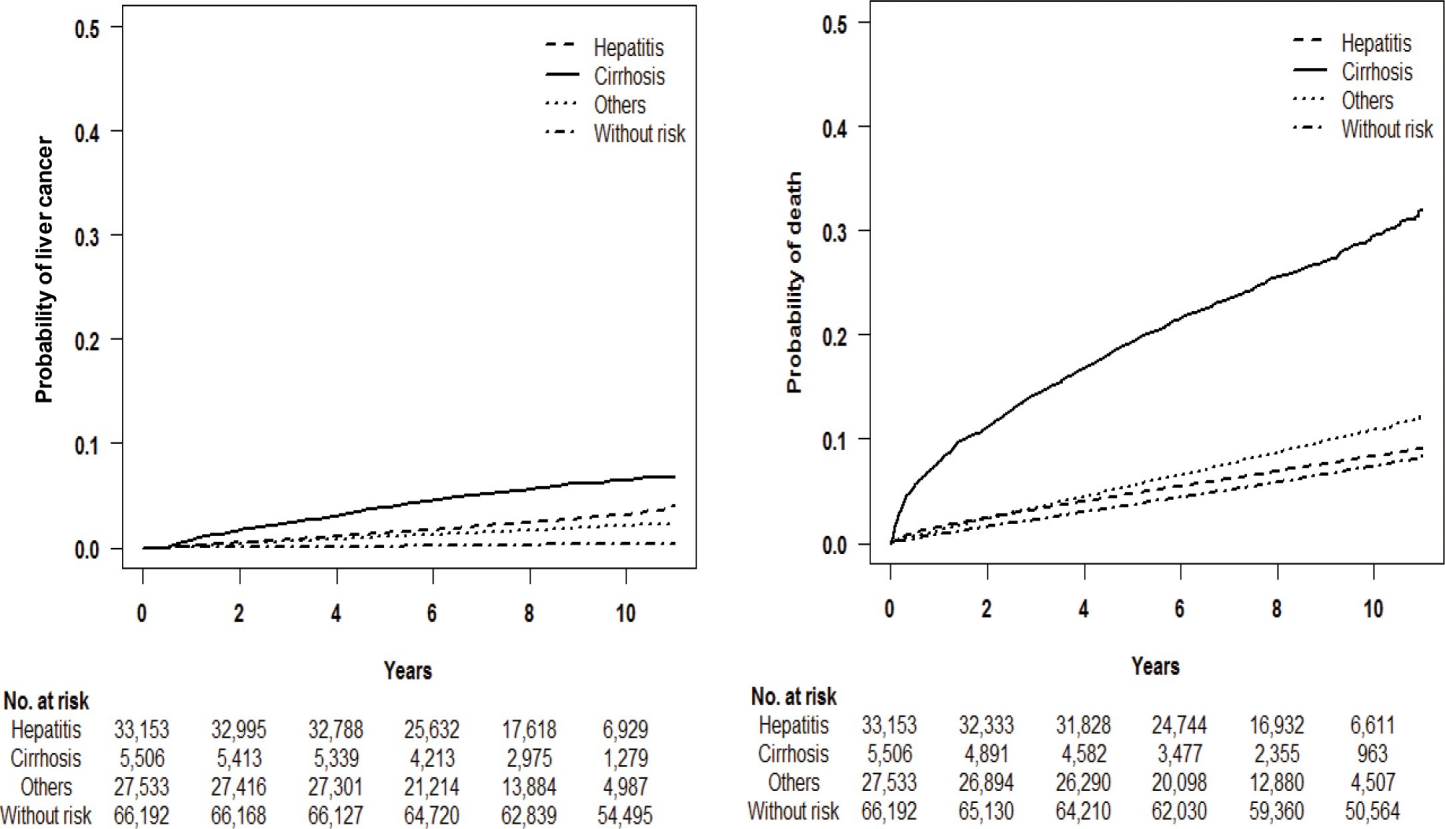
KM curve for liver cancer development and mortality in patients with and without liver disease.

In the liver disease and non-liver disease groups, 1,772 (2.68%) and 210 (0.34%) subjects were diagnosed with liver cancer, respectively within 8 years (median) of the follow-up period. A total of 6,097 (9.21%) and 5,137 (7.76%) subjects from the liver disease and non-liver disease groups, respectively, died with no diagnosis of liver cancer (non-liver cancer-related mortality). Both the probabilities of developing liver cancer and non-liver cancer-related mortality were highest in patients with cirrhosis. The probability of liver cancer development was higher in patients with hepatitis (B, C, or un-identified hepatitis) whereas non-liver cancer-related mortality was higher among patients with other liver diseases (Fig 2).

When the patients with liver disease were categorized by disease type, 29.35% of the patients had HBV and less than 10% had either HCV or hepatitis with no information about hepatitis on B or C in the claims data. Cirrhosis was identified in 9.39% of the patients and 41.60% of the patients had other liver diseases including alcoholic and non-alcoholic liver disease. The total proportion of patients aged at least 40 years was 66.05% and the proportion was low among those with non-alcoholic liver disease (44.71%) and hepatitis B (56.36%). The median follow-up duration was 7.9 years (interquartile range: 5.8–9.6). The incidence of liver cancer was highest in patients with cirrhosis (5.58%) followed by those with hepatitis B (2.72%) and hepatitis C (2.25%) (Table 2).

**Table 2:** Incidence of liver cancer among patients with liver disease.

		Patients with liver disease		≥40 years old		Follow-up		Incidence of liver cancer	
		n	(%)	n	(%)	Median	(IQR)	n	(%)
Total		66,192		43,723	(66.05)	7.9	(5.8, 9.6)	1,772	(2.68)
Hepatitis		33,153	(58.40)	24,322	(62.91)	8.1	(6.0, 9.7)	826	(2.49)
	Hepatitis B	19,952	(29.35)	10,947	(56.36)	8.0	(5.9, 9.7)	543	(2.72)
	Hepatitis C	6,566	(9.74)	4,446	(68.97)	6.6	(4.9, 8.8)	148	(2.25)
	Hepatitis B and C	519	(0.76)	288	(57.14)	6.7	(5.0, 8.5)	5	(0.96)
	Non-specified	6,116	(9.17)	3,471	(57.21)	9.1	(8.0, 10.2)	130	(2.13)
	Cirrhosis	5,506	(9.39)	5,170	(83.16)	7.3	(4.8, 9.5)	307	(5.58)
Others		27,533		19,401	(70.46)	7.8	(5.8, 9.5)	468	(1.70)
	Alcoholic	25,106	(37.93)	18,316	(72.95)	7.8	(5.8, 9.4)	416	(1.66)
	Non-alcoholic	2,427	(3.67)	1,085	(44.71)	8.0	(6.0, 9.6)	41	(1.69)

IQR: interquartile range

### Impact of liver disease on HCC

Cox regression analysis for liver cancer incidence was performed, adjusting for the type of liver disease, sex, age, income level, and comorbidities with competing risk of death (Table 3). Patients with liver disease had an increased relationship with the incidence of liver cancer (hazard ratio [HR]: 8.04; 95% confidence interval [CI]: 7.01–9.22, *p* < 0.0001). When the type of liver disease was introduced in Model 2 instead of the existence of liver disease, cirrhosis had the highest risk (HR: 18.13, 95% CI: 15.24–21.58), followed by hepatitis B (HR: 9.32, 95% CI: 8.00–10.85) (Table 3).

**Table 3:** Cox proportional hazard regression for liver cancer with competing risk of death*.

		Model 1				Model 2			
		HR	95% CI		p-value	HR	95% CI		*p*-value
**Liver disease**	**No**	*Ref*				*Ref*			
	**Yes**	8.04	7.01	9.22	<0.0001				
	Hepatitis					6.36	5.13	7.88	<0.0001
	Hepatitis B					9.32	8.00	10.85	<0.0001
	Hepatitis C					8.21	6.65	10.13	<0.0001
	Hepatitis B and C					3.70	1.52	8.97	0.0004
	Cirrhosis					18.13	15.24	21.58	<0.0001
	Others					5.64	4.82	6.59	<0.0001

*Adjusted for the comorbidities

: hepatocellular carcinoma; HR: hazard ratio; CI: confidence interval.

### Impact of liver disease on liver cancer in subgroups

**Fig 3:**
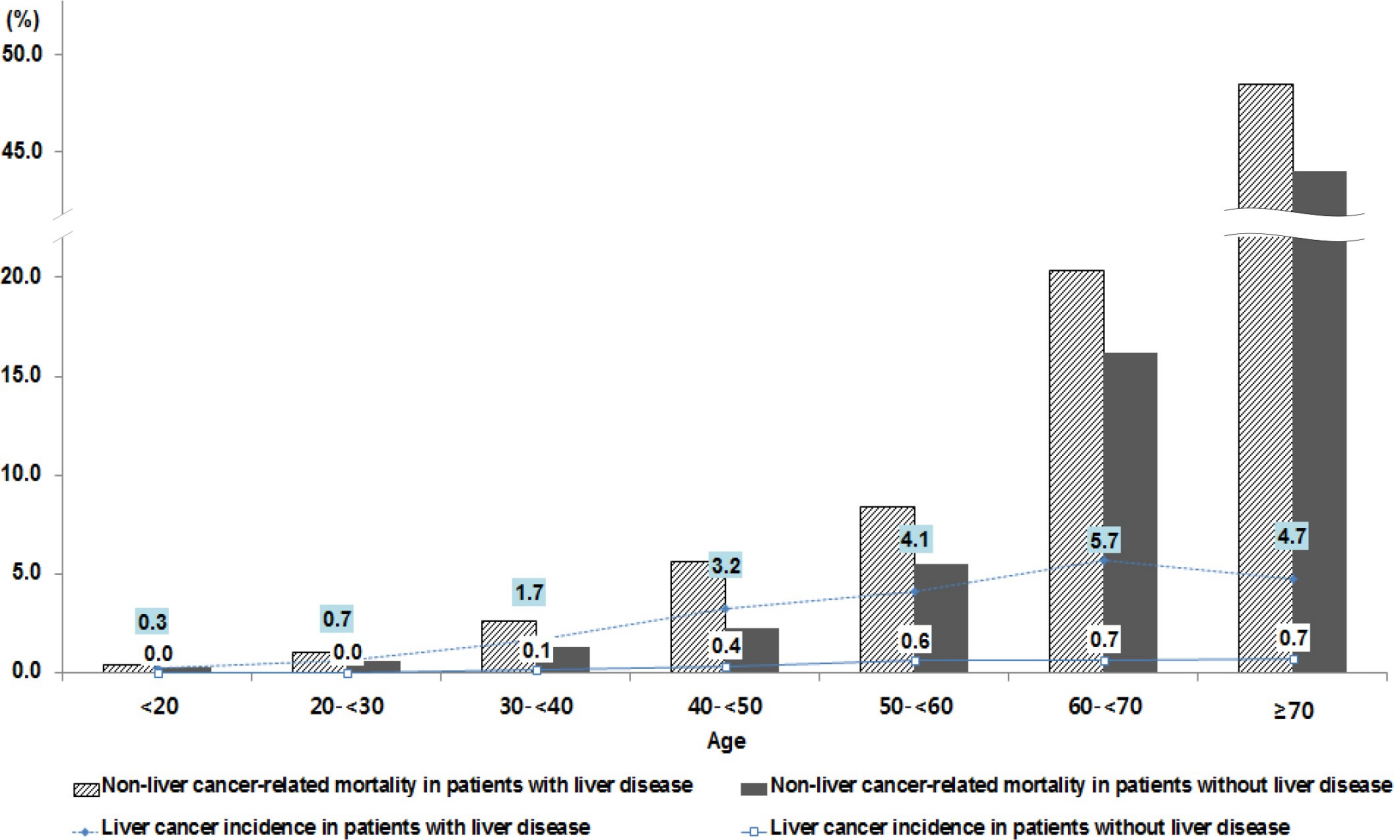
Incidence of liver cancer in patients with and without liver cancer risk stratified by age.

To examine the impact of liver disease on liver cancer in each subgroup, Cox analysis was conducted after stratifying by age and income level based on the insurance type (Table 4). When stratified by income level, the presence of liver disease indicated a very high risk for liver cancer in the Medicaid group (HR: 37.43, 95% CI: 5.16–271.45). When stratified by age, the presence of liver disease was an important risk factor in younger age groups as well as among elderly people. The strength of the association was greater in younger people under 50 years old than in elderly people. The HR for developing liver disease was highest in the 40–49 year age group (HR: 20.08, 95% CI 13.67–29.51), followed by the 30–39 year age group (HR: 18.98, 95% CI: 10.02–35.97) (Table 4). For further understanding, the incidence of liver cancer and non-liver cancer-related mortality were compared by age group and the patterns between young and elderly people were different (Fig 3). Among elderly persons aged 70 years and above, there were 48.5 and 43.9 non-liver cancer-related deaths per 100,000 in patients with and without liver disease, respectively, and the liver cancer incidence was 4.7 and 0.7 per 100,000, respectively. The rate of non-liver cancer-related mortality was much higher than that for liver cancer incidence. However, among younger people, the non-liver cancer-related mortality rate was low in both patients with and without liver disease (1.0 vs. 0.6 for the 20–29 year age group and 2.6 vs. 1.3 for the 30–39 year age group, respectively) but the liver cancer incidence was approximately 10–20 times higher among people with liver disease (0.7 vs. 0.01 in the 20–29 year age group and 1.7 vs. 0.1 in the 30–39 year age group) (Fig 3).

**Table 4:** Hazard ratio for liver cancer in patients with and without liver disease after stratification of age and income level.

			Patients with liver disease		Matched patients without liver disease		HR* (95% CI)	
			n (liver cancer cases)	(%)	n (liver cancer cases)	(%)		
**Total**			1,772	(2.68)	210	(0.34)	8.04	(7.01, 9.22)
**Age**	0–19		6	(0.21)	1	(0.03)	5.16	(0.63, 42.33)^1)^
	20–29		40	(0.56)	5	(0.07)	11.14	(4.26, 29.13) ^1)^
	30–39		140	(1.13)	11	(0.09)	18.98	(10.02, 35.97) ^1)^
	40–49		417	(2.50)	28	(0.17)	20.08	(13.67, 29.51) ^1)^
	50–59		448	(3.40)	58	(0.44)	11.26	(8.52, 14.89) ^1)^
	60–69		352	(3.91)	82	(0.91)	5.7	(4.44, 7.33) ^1)^
	**≥**70		187	(3.80)	67	(1.36)	3.53	(2.65, 4.70) ^1)^
**Level of income based on type of insurance**	NHI district subscriber	<50%	336	(3.12)	45	(0.42)	9.55	(6.96, 13.11) ^2)^
		**≥**50%	457	(2.56)	58	(0.33)	9.76	(7.39, 12.88) ^2)^
	NHI employee subscriber	<50%	319	(2.21)	54	(0.37)	7.62	(5.62, 10.32) ^2)^
		**≥**50%	443	(1.99)	94	(0.42)	5.95	(4.73, 7.49) ^2)^
	Medicaid		35	(3.68)	1	(0.11)	37.43	(5.16, 271.45) ^2)^

*HR in matched patients with and without liver disease.

^1)^ HR was estimated using a Cox proportional hazard model with competing risk of death adjusted for sex, income level, and comorbidities.

^2)^ HR was estimated using a Cox proportional hazard model with competing risk of death adjusted for sex, age, and comorbidities.

HR: hazard ratio; CI: confidence interval; NHI, National Health Insurance.

## Discussion

This study compared the incidence of liver cancer in patients with and without liver disease to examine the impact of liver disease on liver cancer incidence in Korean population. Approximately, 2.7% of patients with liver disease developed liver cancer within 8 years (median) of the follow-up period, which was 8-fold higher compared with those without liver disease (0.34%). In other words, the annual incidence of liver cancer was estimated at approximately 335/100,000 (2.7% for 8 years of median follow-up period = 335/100,000 per year) and 42.5/100,000 (0.34% for 8 years of median follow-up period = 42.5/100,000 per year) among patients with and without liver disease, respectively. It may not be possible to directly compare our study findings with those of previous study because there were no studies on the liver cancer incidence in respective patients with liver disease and without liver disease. However, it was reported that the crude incidence of liver cancer, based on the cancer registry data of South Korea, was 28.2/100,000 in 1999 and 31.9/100,000 in 2014, respectively [14]. Considering that most of the Korean population generally had no liver disease and we had more middle-aged and elderly people in the data compared to the general population, the liver cancer incidence of the patients without liver disease in our data was comparable with the crude incidence from the cancer registry.

The causal relationship between liver cancer and liver diseases such as cirrhosis, hepatitis B, and hepatitis C has been well documented in previous studies. However, previous studies in South Korea investigated the risk factors among only cancer patients, instead of comparing with patients with liver diseases or using a cohort design. This study was able to identify the magnitude of the risk in age or sex- matched patients with liver disease compared to those without liver disease [15–18].

In terms of baseline liver disease, the risk for liver cancer was 15, 12, and 9 times higher in patients with cirrhosis, hepatitis B, and hepatitis C, respectively, than in those without liver disease. This result was consistent with other previous studies. Cirrhosis was known to be associated with a high risk of liver cancer [19–22]. In another study based on Korean population, the HBV- and HCV-related HCC incidence per 100,000 persons was 20.8 and 4.9, with a lower survival rate in patients with HBV-related HCC than in patients with HCV-related HCC 22. Similar to results from South Korea, a meta-analysis of 13 studies conducted in China, which has a higher prevalence of HBV, showed that the odds ratio (OR) for the HCC incidence due to HBV infection was 58.01 (95% CI: 44.27–71.75), whereas the OR due to HCV infection was 2.34 (95% CI: 1.20–3.47) and the OR due to HBV/HCV double infection was 11.39 (95% CI: 4.58–18.20) [21].

In this study, the risk for developing liver cancer was lower in patients co-infected with HBV/HCV (HR: 4.93, 95% CI: 4.33–5.91) than in patients with only HBV (HR: 12.48, 95% CI: 10.71–14.56) or HCV (HR: 9.20, 95% CI: 7.45–11.37). Although some studies showed that the risk for HCC in patients with both HBV and HCV is higher than in those with HBV or HCV alone, the Chinese study reported a lower risk than that of HBV alone [21]. The lower risk for co-infection in our study might come from the limitation of using claims data. It is possible that only one type of hepatitis was recorded when another type of hepatitis occurred later but was omitted from the claims data, leading to an underestimation of the development of liver cancer in patients with HBV/HCV co-infection.

Age was the main risk factor for liver cancer. Most studies mentioned that HCC increased as age increased [19, 23] as shown in our study. However, stratification by age group revealed that the risk for liver cancer in patients with liver disease was much higher than in those without liver disease in patients younger than 50 years. This trend was more distinct when comparing liver cancer incidence with non-liver cancer-related mortality by age. Among elderly people with or without liver disease, the non-liver cancer mortality was high and the liver cancer incidence was low. However, the presence of liver disease in younger age seems to make a bigger impact on the incidence of liver cancer. In Korea, although perinatal infection rate has decreased to 0.1% since 2002, it is still the main cause of hepatitis with particularly HBV infection leading to HCC. In a previous study, the median survival of HCC due to HBV was lower than that due to HCV (2.17 vs. 1.34 years). The authors presumed that one of the reasons was the late detection of HCC related to HBV, which was more prevalent among younger people 22. Nonetheless, the NLCSP in South Korea was conducted among people older than 40 years old. For the continuous and systemic management of younger people with liver disease, lowering the age of eligibility for the NLCSP may be considered.

The liver cancer risk in patients with liver disease was highest in the Medicaid subgroup. In a previous study, the HCC incidence rate ratio (IRR) was high in the lower three income levels (IRR: 1.24; 95% CI: 1.07–1.42; IRR: 1.18; 95% CI: 1.03–1.36; IRR: 1.16; 95% CI: 1.00–1.34, respectively) [24]. In our study, even though the number of subjects in the Medicaid group was small, the risk for people with liver disease was 37 times higher than in those without liver disease within this group. These data highlighted the importance of management to prevent liver disease progressing to liver cancer in the Medicaid group as well as the detection of liver cancer. Because the Medicaid group and people whose income level was lower than 50th percentile already had no copayments in the NLCSP, the cost for liver cancer detection would not be borne by lower-income people. However, the treatment for patients with liver disease for prevention of liver cancer would be differentiated by income level. Furthermore, in patients with a lower income level, the HCC treatment cure rate was also lower in a previous study 20. Liver transplantation, one of the curative treatment modalities, would only be possible for patients with high income. Therefore, public health efforts aimed at preventing the development of liver cancer in Medicaid recipients with liver disease in addition to the HCC surveillance program would be required.

### Limitations

This study has the limitation of using the NHI claims database, which was established for the purpose of reimbursements, and the claimed diagnosis may not fully match the clinical diagnosis. To overcome this limitation, we identified liver cancer patients as those with a liver cancer diagnostic code on an inpatient record. Second, we could not distinguish between HCC and other liver cancers because the claim database did not have enough clinical information to distinguish two diseases. However, most liver cancers would be regarded as HCC 4. Third, the comparability of people with or without liver disease was not easy owing to their different baseline characteristics. Therefore, we matched the two groups according to social and demographic information. We also adjusted for differences in the clinical information between the two groups.

### Conclusion

This study confirmed a higher risk of liver cancer in the Korean population with liver disease, and the risk for liver cancer by liver disease in younger people or in Medicaid recipients should be highlighted. It is likely that attention to the development of liver cancer in persons younger than 50 years and prevention efforts aimed at decreasing the liver cancer incidence among Medicaid recipients are needed.
